# Physicochemical data of oleic acid-poloxamer organogel for intravaginal voriconazole delivery

**DOI:** 10.1016/j.dib.2019.104180

**Published:** 2019-06-24

**Authors:** Samyr M. Querobino, Naially C. de Faria, Aryane A. Vigato, Bruna G.M. da Silva, Ian P. Machado, Maricilia S. Costa, Fanny N. Costa, Daniele R. de Araujo, Carlos Alberto-Silva

**Affiliations:** a Universidade do Estado de Minas Gerais - UEMG, Av. Juca Stockler, 1130, Bairro Belo Horizonte, Passos, 37900-106, MG, Brazil; b Natural and Human Sciences Center, Federal University of ABC (UFABC), Av. dos Estados, n° 5001, Bloco A, Torre 3, Lab 503-3, Santo André, SP, Brazil; c Instituto de Pesquisa e Desenvolvimento – IP&D, Universidade do Vale do Paraíba (UNIVAP), Av. Shishima Hifumi, n° 2911, São José dos Campos, SP, Brazil; d Department of Fundamental Chemistry, Institute of Chemistry, University of São Paulo, 05588-000, SP, Brazil; e Natural and Human Sciences Center, Federal University of ABC (UFABC), Rua Arcturus, n° 03, Bloco Delta, São Bernardo do Campo, 09606-070, SP, Brazil

**Keywords:** Voriconazole, Poloxamer, Vaginal release, Organogel, Sodium alginate, Oleic acid

## Abstract

Functional polymeric nanoparticles have attracted attention for different biomedical applications, including drug delivery. Poloxamers (PL), a synthetic copolymers of poly(ethyleneoxide)-b-poly(propylene oxide)-b-poly(ethylene oxide), that exhibit thermoreversible behavior in aqueous solutions. Physicochemical properties of Oleic Acid-Poloxamer (OA-PL) organogel for intravaginal controlled Voriconazole (VRC) delivery were assessed using three different oils (isopropyl myristate - IPM, isopropyl palmitate - IPP, and oleic acid – OA, in order to select the most suitable oil phase for increasing the solubility of the drug and its dispersion in the final aqueous phase. Organogel structural organization was assessed by VRC partition coefficient, differential scanning calorimetry (DSC), rheological analysis, and drug release assay. These data are complementary to the research article entitled “Sodium alginate in oil-poloxamer organogels for intravaginal drug delivery: influence on structural parameters, drug release mechanisms, cytotoxicity and *in vitro* antifungal activity” - Materials Science and Engineering: C, 2019. 99: p. 1350–1361.

**Table:** Specifications table

Subject area	*Pharmaceutical Technology*
More specific subject area	*Controlled drug delivery system.*
Type of data	*Table and figure*
How data was acquired	*Partition coefficient; Differential scanning calorimetry (DSC); Rheometer; Drug release; rSpace for Kinexus software; GraphPad Prism 7.0 software*
Data format	*Analyzed*
Experimental factors	*The poloxamer solution (188 and 407 – 15 and 30 % wt) was prepared according to the cold method. After was add the organic phase composed by oleic acid and voriconazole (*5 mg/mL*)*
Experimental features	***Partition coefficient:****Voriconazole (VRC) partition coefficient determination was assessed using three different oils (isopropyl myristate - IPM, isopropyl palmitate - IPP, and oleic acid - OA). The partition coefficient (P) was calculated as the ratio between the VRC concentrations in the aqueous and organic phases, expressed as log P.****DSC:*** 20 mg *of the organogel samples were placed in a sealed aluminum pan, and analyzed according to three successive thermal cycles of heating and cooling (0 to 50 °C), at a rate of* 5 °C/min*.****Rheology:****Rheological parameters were obtained with cone-plate geometry. Organogel samples (*1 mL*) were analyzed using a temperature range from 8 to 80 °C, a frequency of* 1 Hz*, and shear stress of* 2 Pa*.****Drug release:****The drug release was performed using membraneless model, and VRC release profiles were analyzed according to Zero-order, Higuchi, Hixson-Crowell and Korsmeyer−Peppas models equations*
Data source location	*Federal University of ABC, Av. dos Estados, n° 5001, Bloco A, Torre 3, Lab 503–3, Santo André, SP, Brazil.*
Data accessibility	*The data are with this article.*
Related research article	*Querobino, S.M.* et al.*, Sodium alginate in oil-poloxamer organogels for intravaginal drug delivery: Influence on structural parameters, drug release mechanisms, cytotoxicity and in vitro antifungal activity. Materials Science and Engineering: C, 2019. 99: p. 1350–1361*[1]

**Table:** 

**Value of the data** • The data presented here could be useful to better understand the poloxamer-organogel behavior and their future application as a promising drug delivery system for intravaginal candidiasis treatment. • The OA-PL organogel showed high stability, being a potential controlled drug delivery system to be explored. • OA nucleus induced organization of the systems by interacting with the PPO blocks in the micellar core. • OA-PL organogels could provide the basis for new VRC delivery systems for use in future vaginal applications.

## 1 Data

**Fig. 1:**
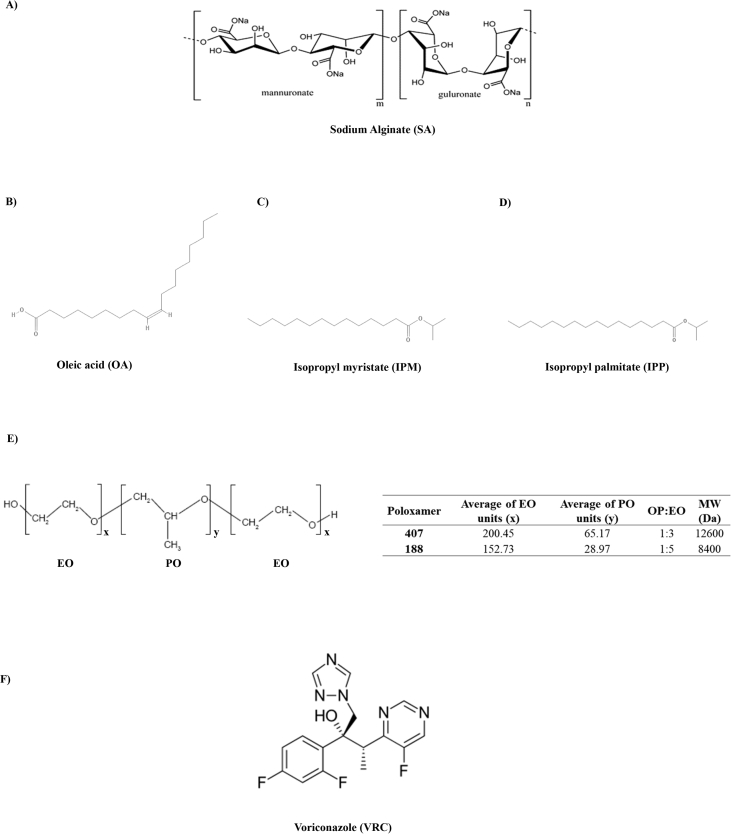
Chemical structures of voriconazole (A), sodium alginate (B), the organic solvents tested as the oil phase [oleic acid (C), isopropyl myristate (D), and isopropyl palmitate (E)], and poloxamer (F).

**Fig. 2:**
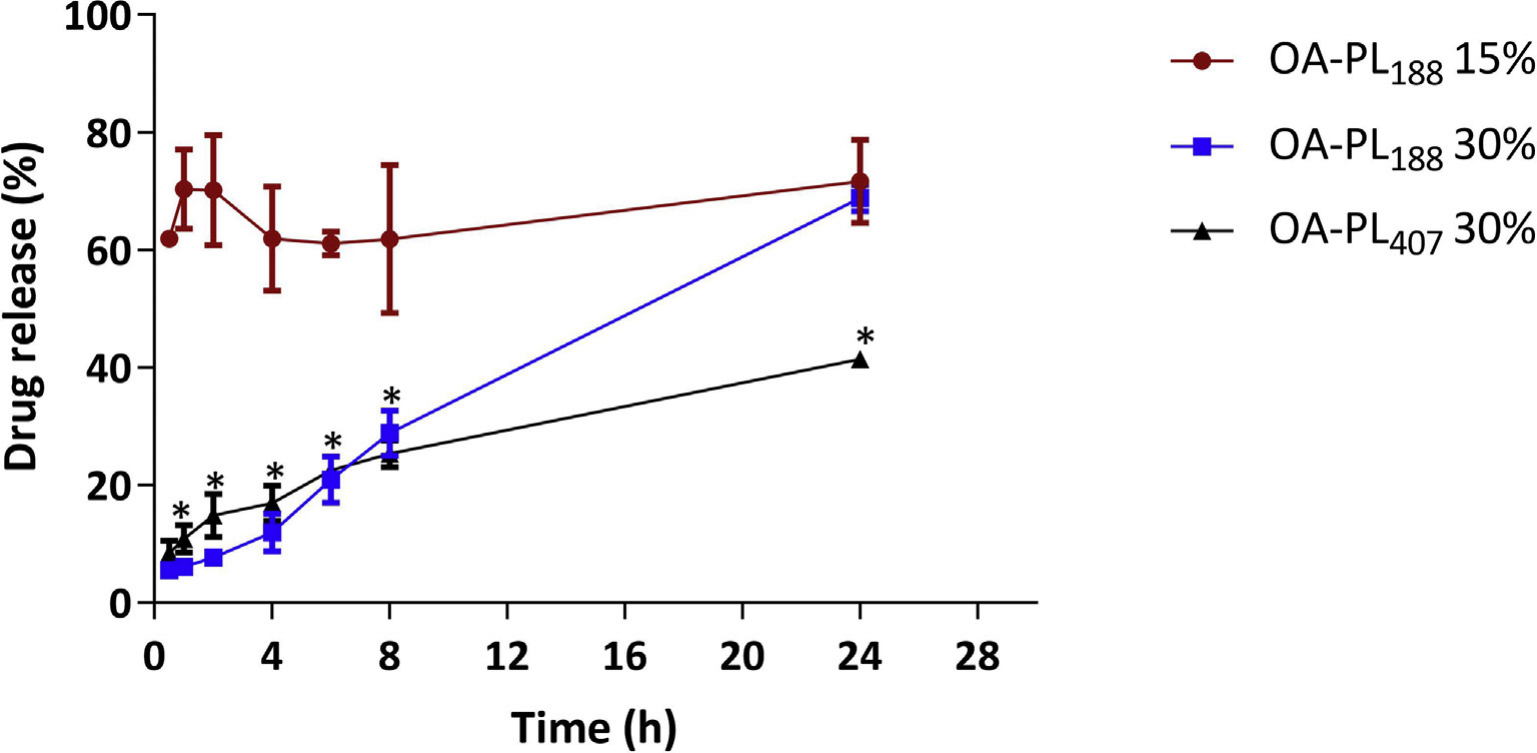
Voriconazole *in vitro* release profiles from OA-PL188 15 and 30% and OA-PL407 30%. The values are expressed as mean ± standard deviation from three independent experiments in triplicate, with analysis using one-way ANOVA followed by Tukey's post-test. *p < 0.05. OA - oleic acid; PL_407_ - poloxamer 407, PL_188_ - poloxamer 188.

Poloxamers (PL) exhibit thermoreversible behavior in aqueous solutions [2], [3], [4], [5], [6], [7], [8], [9], and it has been utilized for different biomedical applications, including drug delivery [10], [11], [12]. Fig. 1 illustrates all chemical structures of formulations compounds. Table 1 shows the highest log P_VRC_ value for OA:water (1.52), followed by IPM:water (0.24) and IPP:water (0.21). In PL formulations, the micellization temperature peaks (Tm) extending over ∼12 °C in the DSC data. Table 2 demonstrates that OA-PL_188_ 30% T_sol-gel_ was in the ranges ∼39 °C similar results were observed in the presence of simulated vaginal fluid (SVF). The OA-PL_188_ formulations were unstable, since the elastic modulus values decreased following temperature variation, resulting in low G’/G″ ratios (from 0.2 to 5.3) and low viscosities (from 4.3 to 0.02 mPa s .10^3^). For OA-PL_407_ 30% in the presence or absence of SVF was lower than 8 °C. This formulation was stable presenting high G’/G” ratios (from 9 to 25) at 37 °C in the absence of SVF and 29.1–30.9 in the presence of SVF. Also, high viscosity values were observed for OA-PL_407_ 30% range from 1027 to 2191 m. Pas.10^3^. Fig. 2 demonstrates that OA-PL_407_ was released until 8 h and reaching lower release percentages (from 30%) than that obtained for OA-PL_188_ (p < 0.01). Table 3 shows that the formulations composed of OA-PL_407_ presented high correlation coefficient values obtained for the models Higuchi, similar to observed for OA-PL_188_ 30%, values obtained for the models Higuchi (R^2^ = 0.96) and Zero-order model (R^2^ = 0.98). The OA-PL_188_ 15% did show good correlation coefficient in any Kinect model tested.

**Table 1:** Temperatures (T) and enthalpy variations (ΔH_m_) associated with the phase transitions for the PL-SA-OA-based organogels.

Formulation		Additives	T_onset_ (°C)	T_m_ (°C)	T_endset_(°C)	ΔH_m_(J.g^−1^)
OA-PL_188_	15%	–	9.2	12.9	17.5	4.5
		VRC	5.6	13.4	18.4	35.5
	30%	–	8.8	11.7	13.5	0.7
		VRC	8.2	12.2	15.8	3.4
OA-PL_407_	30%	–	9.6	13.2	17.2	8.4
		VRC	8.9	11.6	14.7	0.9

Note: T_onset_: initial micellization temperature; T_m_: micellization temperature peak; T_end_: final micellization temperature. Enthalpy variation (ΔH_m_) refers to the micellization process. SA: sodium alginate; OA: oleic acid; VRC: voriconazole; PL: poloxamer.

**Table 2:** Rheological parameters for OA-PL_407_ and OA-PL_188_ in the absence (−) and presence (+) of simulated vaginal fluid (SVF).

Simulated vaginal fluid (SVF)	Formulation		Additives	T (sol-gel) °C	25 °C				37 °C			
					G' (Pa)	G'' (Pa)	G'/G''	η* (mPas.s) x 10³	G' (Pa)	G'' (Pa)	G'/G''	η* (mPas.s) x 10³
**(−)**	PL _188_	15%	–	Nd	0.16	0.04	4	0.02	0.16	0.03	5.3	0.02
			VRC	Nd	0.16	0.06	2.6	0.02	0.16	0.05	3.2	0.02
		30%	–	39.6 ± 0.9	0.17	0.31	0.5	0.05	2.1	13.2	0.2	2.1
			VRC	39.4 ± 0.8	0.17	0.39	0.4	0.07	5.7	28.8	0.2	4.3
	PL _407_	30%	–	<8	6784	784	8.7	1087	6429	712	9	1027
			VRC	<8	14800	385	38.5	2356	13760	555	25	2191
**(+)**	PL _188_	15%	–	Nd	0.03	5.4	0.005	23.9	0.02	7.8	0.003	0.02
			VRC	Nd	0.03	4.3	0.007	23.3	0.02	6.1	0.003	0.02
		30%	–	39.7 ± 0.7	0.2	0.2	1	0.04	1.5	8.9	0.16	1.4
			VRC	39.2 ± 0.6	0.2	0.2	1	0.03	0.8	5.4	0.14	0.9
	PL _407_	30%	–	<8	13480	350	38.5	2147	12240	420.5	29.1	1948
			VRC	<8	9210	244	37.7	1466	8177	264	30.9	1302

Note: The values refer to the G′ (elastic) and G″ (viscous) moduli, and viscosity (η), at 25 and 37 °C, and the sol-gel transition temperatures (Tsol-gel).

**Table 3:** Drug release constants and correlation coefficients obtained for organogel formulations.

Formulation		Higuchi		Zero order		Hixson-Crowell		Korsmeyer-Peppas		
		R^2^	K_0_ (%.h^−1/2^)	R^2^	K_0_ (%.h^−1^)	R^2^	K_HC_ (%.h^−1/3^)	R^2^	K_KP_ (%.h^−n^)	n
PL_407_	30%	0.96	7.8 ± 0.4	0.90	1.3 ± 0.1	0.79	0.05 ± 0.01	0.91	10.1 ± 1	0.4 ± 0.03
PL_188_	15%	0.03	0.99 ± 1.2	0.06	0.3 ± 0.3	0.06	0.01 ± 0.01	0.006	64 ± 1	0.01 ± 0.02
	30%	0,96	7,8 ± 0,4	0,98	2.7 ± 01	0.91	0.1 ± 0.01	0.90	6.1 ± 1	0.68 ± 0.06

Data presented as mean ± S.D. (n = 3/formulation).

## 2 Experimental design, materials, and methods

### 2.1 Voriconazole partition coefficient determination

For partition coefficient (P) determination, VRC (5 mg/mL, 0.5%) was solubilized in 1 mL of three different organic phases (IPM, IPP, and OA) (Fig. 1), followed by mixing with an equal volume (1 mL) of ultrapure water. The solutions were then homogenized and stored at 25 °C for 24 h. The samples were centrifuged (1917 *x g* for 15 min) to ensure complete separation of the two phases. The organic phase was removed and VRC present in the aqueous phase was quantified by HPLC. The P value was used as a parameter for selection of the most suitable oil phase for solubilizing VRC and preparing the organogel formulations. The P was calculated as the ratio between the VRC concentrations in the aqueous and organic phases, expressed as log P. T the chemical structures of these three oils, OA (C_18_H_34_O_2_) presents a carbon chain length intermediate between those of IPP (C_19_H_38_O_2_) and IPM (C_17_H_34_O_2_), while the presence of available carboxyl groups in the OA structure enables the formation of hydrogen bonds with electronegative atoms (nitrogen and fluorine) present in the VRC chemical structure (Fig. 1). On the other hand, IPM and IPP are esters that possess bulky isopropyl radicals that reduce the possible interactions with the VRC molecule. Therefore, OA was selected as the oil phase for all the organogel formulations.

### 2.2 Organogel sample formulation

The aqueous phase (AP) composed of PL407 or PL188 (at 15 or 30 wt%), was dispersed in 20 mM citrate buffer (CB) at pH 4.7, with magnetic stirring at 450 rpm and 4 °C until complete dissolution until a homogeneous hydrogel was obtained. Sodium benzoate (0.25 wt%) was added to all formulations as a preservative. VRC was solubilized in an oil phase (OP) composed of oleic acid (selected according to the results of the partition coefficient assays), followed by homogenization with the PL aqueous phase, using a 1:4 (v/v) OP:AP ratio. The final VRC concentration was 5 mg/mL.

### 2.3 Differential scanning calorimetry

Differential scanning calorimetry (DSC) experiments were performed with Polyma DSC system (Netzsch, Germany). Organogel samples (20 mg) were placed in a sealed aluminum pan, and analyzed according to three successive thermal cycles of heating and cooling (0–50 °C), at a rate of 5 °C/min, using an empty pan as a reference [3]. Data were expressed in thermograms represented by heat flux (J/g) versus temperature (°C). The micellization temperature peaks (Tm) PL formulations were over ∼12 °C (Table 1) in the DSC data. Pluronics are triblock copolymers of poly(ethylene oxide)–poly(propylene oxide)–poly(ethylene oxide) (abbreviated by PEO–PPO– PEO hereafter). Their amphiphilic nature is simply due to the combination of the hydrophobic PPO segment and the hydrophilic PEO segments [13]. The OA-PL407 30% shown higher micellization temperature than OA-PL188 30%, PL of smaller molecular weight form micelles more difficulty at higher concentrations and temperatures, due to more structure barrier of micellization process. The effect of PEO:PPO ratio in the micellization process is more difficult to form micelles for more hydrophilic Pluronics. The VRC addition in the OA-organogel reduced the micellization temperature in the formulations evaluated.

### 2.4 Rheological properties measurement

Rheological parameters (elastic modulus - G′, viscous modulus - G″, and viscosity - η*) were obtained using assays performed with a Kinexus Lab rotational rheometer (Malvern Instruments Ltd., UK) with cone-plate geometry. Organogel samples (1 mL) were analyzed using a temperature range from 8 to 80 °C, frequency of 1 Hz, and shear stress of 2 Pa, for the sol-gel transition temperature (Tsol-gel) determination. In order to simulate the application of the organogels and their dilution in the SVF, samples (1 mL) were placed on a vaginal applicator and applied to the rheometer plate, followed by the addition of SVF (375 μL) [14]. SVF was prepared as described by Owen and Katz [15] containing the following components: NaCl, 3.51 g/L (Synth); KOH, 1.40 g/L (Synth); Ca(OH)2, 0.222 g/L (Synth); bovine serum albumin, 0.018 g/L (Sigma-Aldrich); lactic acid, 2.00 g/L (Synth); acetic acid, 1.00 g/L (Sigma-Aldrich); glycerol, 0.16 g/L (Sigma-Aldrich); urea, 0.4 g/L (Vetec, Rio de Janeiro, Brazil); glucose, 5.0 g/L (Sigma-Aldrich). The pH of the SVF was adjusted to pH 4.2. The data were analyzed using rSpace for Kinexus software. The presence of SVF resulted in a decrease of the final PL concentration, changing the T_sol-gel_, G’, and G” parameters. In addition, the co-solutes (ions, proteins, and electrolytes) present in the SVF [14] can interact with OA-PL_407_ and OA-PL_188_, which was a more hydrated system, due to the high PEO units present in their structure (1:5 PPO:PEO ratio).

### 2.5 Drug release and release kinect models

The drug release was performed using membraneless model. We used a two-compartment system composed of a separate glass cell and inserted in a receptor compartment. The dissolution medium was SVF (37 °C). At regular intervals from 0.5, 1, 2, 4, 6, 8–24 h, 1 mL of the receptor medium was withdrawn and the drug content was analyzed by HPLC and expressed as release percentage against time. The VRC release profiles were analyzed according to Zero-order, Higuchi, Hixson-Crowell and Korsmeyer−Peppas models equations.

### 2.6 Statistical analyses

The data were presented as means ± S.D. of three independent experiments (n = 3), performed in triplicate. The statistical technique used was one-way analysis of variance (ANOVA) followed by the Tukey-Kramer post-hoc test for multiple comparisons. A standard significance level of p < 0.05 was used. The analyses were performed with GraphPad Prism 7.0 software (GraphPad Software, Inc., La Jolla, CA, USA).
